# Design and Analysis of Porous Elastomeric Polymer Based on Electro-Mechanical Coupling Characteristics for Flexible Pressure Sensor

**DOI:** 10.3390/polym16050701

**Published:** 2024-03-04

**Authors:** Yingxuan Bu, Jian Wu, Zheming Zhang, Qiandiao Wei, Benlong Su, Youshan Wang

**Affiliations:** 1 Center for Rubber Composite Materials and Structures, Harbin Institute of Technology, Weihai 264209, China; buyingxuan@126.com (Y.B.); 2200790420@stu.hit.edu.cn (Z.Z.); weiqiandiao@gmail.com (Q.W.); subenlong@hit.edu.cn (B.S.); wangys@hit.edu.cn (Y.W.); 2 National Key Laboratory of Science and Technology on Advanced Composites in Special Environments, Harbin Institute of Technology, Harbin 150090, China

**Keywords:** porous elastomeric polymer, capacitive flexible sensor, finite element method, electro-mechanical coupling

## Abstract

Elastomeric polymers have gained significant attention in the field of flexible electronics. The investigation of the electro-mechanical response relationship between polymer structure and flexible electronics is in increasing demand. This study investigated the factors that affect the performance of flexible capacitive pressure sensors using the finite element method (FEM). The sensor employed a porous elastomeric polymer as the dielectric layer. The results indicate that the sensor’s performance was influenced by both the structural and material characteristics of the porous elastomeric polymer. In terms of structural characteristics, porosity was the primary factor influencing the performance of sensors. At a porosity of 76%, the sensitivity was 42 times higher than at a porosity of 1%. In terms of material properties, Young’s modulus played a crucial role in influencing the performance of the sensors. In particular, the influence on the sensor became more pronounced when Young’s modulus was less than 1 MPa. Furthermore, porous polydimethylsiloxane (PDMS) with porosities of 34%, 47%, 67%, and 72% was fabricated as the dielectric layer for the sensor using the thermal expansion microsphere method, followed by sensing capability testing. The results indicate that the sensor’s sensitivity was noticeably influenced within the high porosity range, aligning with the trend observed in the simulation.

## 1. Introduction

Flexible sensors based on elastomeric polymers have gained significant interest across a range of fields such as robotics [1,2], healthcare [3,4], traffic monitoring [5], human motion detection [6,7], and intelligent tire technology [8,9,10] owing to their exceptional flexibility and ductility. Flexible capacitive pressure sensors stand out due to their stability and low power consumption [11,12]. The structure is primarily composed of three components: a top electrode, a dielectric layer, and a bottom electrode. The dielectric layer undergoes deformation when subjected to external force, leading to a change in the distance between the electrodes and, consequently, a variation in capacitance. The pressure value can be determined by analyzing the relationship between pressure and the capacitance changes. Therefore, it is of paramount importance to investigate the electro-mechanical response relationship between the dielectric layer and flexible sensor for sensor design. Elastomeric polymers, such as polydimethylsiloxane (PDMS) [13], poly(vinylpyrrolidone) (PVP) [14], poly(2-hydroxyethyl methacrylate) (pHEMA) [15], poly-methyl-methacrylate (PMMA) [16], polyurethane (TPU) [17], and Ecoflex [18], are commonly employed in the fabrication of dielectric layers. Nevertheless, flexible capacitive pressure sensors utilizing bulk elastomeric polymers as the dielectric layer face challenges in achieving sufficient sensitivity. To address this issue, various microstructures are incorporated into the elastomeric polymer, such as micropatterns [19], spacers [20], nanowires [21], pores [22], and textile structures [23], which improve the compressibility of the material and consequently, enhance the sensitivity of the sensor [24]. Porous structures have gained significant research attention due to their ability to offer enhanced performance characteristics, including low density and a large surface area-to-volume ratio [25].

To improve the sensitivity of porous flexible capacitive pressure sensors, some researchers are dedicated to increasing the porosity of elastomeric polymers. Masihi et al. [26] used the carbon dioxide (CO_2_) foaming method to fabricate a high-porosity PDMS dielectric layer. This approach enhanced deformability, thereby enhancing the sensor’s sensitivity. In addition, researchers have added fillers such as carbon black (CB) [27], carbon nanotubes (CNTs) [28], graphene (GR) [29], and barium titanate (BaTiO_3_) [30] into elastomeric polymers. This practice significantly increases the dielectric constant of the polymer, consequently improving its sensitivity. Other researchers have employed low-modulus materials such as Ecoflex [31] to fabricate dielectric layers that exhibit increased deformation under the same pressure levels, leading to improved sensitivity. Additionally, adjusting the dimensions and shape factors of the elastomeric polymer, along with the Poisson’s ratio, can also affect the performance of the sensor [32]. These methods improve the sensitivity of the sensor by either enhancing its deformation capability or improving the electrical properties of the elastomeric polymer. However, further investigation is needed to explore the relationship between the structural and material characteristics of the elastomeric polymer and the sensitivity of the sensor. Furthermore, during the compression process, the stress–strain curve of porous elastomeric polymer generally demonstrates three distinct stages [33]. The initial stage is characterized by linear elastic deformation, during which the modulus remains constant. The second stage is the plateau phase, characterized by minimal stress variation with increasing strain. The final stage is the compact consolidation phase, where contact occurs between pore walls, resulting in a gradual increase in the modulus of the porous elastomeric polymer. The initial stage of linear deformation holds significant importance for applications of flexible sensors. During this stage, sensors demonstrate exceptional linearity and stability, and high sensitivity.

This study focused on the initial linear elastic deformation stage of porous flexible sensors. We investigated the impact of the structural characteristics of the porous elastomeric polymer, including porosity, pore radius, major-to-minor axis ratio of elliptical pores, and rotation angle of the elliptical pores, as well as the material properties, such as Young’s modulus and relative dielectric constant, on the sensor’s sensitivity. Through conducting a two-dimensional (2D) finite element analysis, we investigated the trends in how these factors influenced the sensitivity of the sensor. By adjusting the PDMS-to-curing agent ratio to 10:1, 20:1, and 40:1, we prepared PDMS with a different moduli as the dielectric layer. Subsequently, within a range of 0–20 kPa, we compared the relative capacitance changes and sensitivity of the sensor. Finally, porous PDMS with porosities of 34%, 47%, 67%, and 72% was fabricated using the thermal expansion microsphere method for the dielectric layer of flexible capacitive pressure sensors. We compared the relative capacitance changes and sensitivity of sensors with different porosities within the range of 0–20 kPa.

## 2. Materials and Methods

### 2.1. Finite Element Model

**Figure 1:**
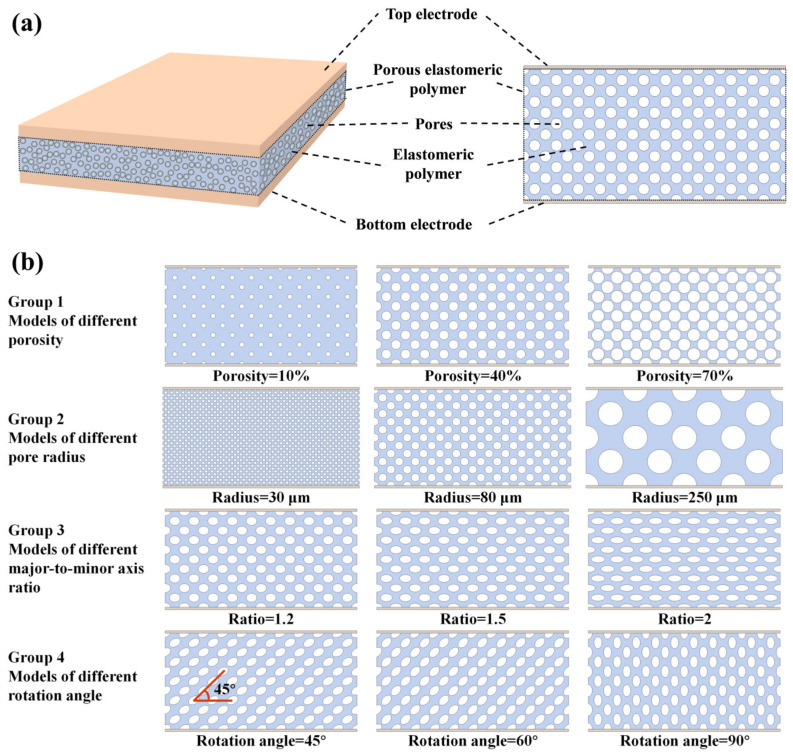
Finite element simulation models. (**a**) 2D finite element model of the sensor structure. (**b**) Sensor structure models for different porous elastomer polymer structures.

The structure of the sensor is illustrated in Figure 1a. To improve computational efficiency, the sensor’s structure was approximated as a 2D FEM. This simulation model consisted of a top electrode, a porous elastomeric polymer, and a bottom electrode. In the 2D model, the white circular region was identified as the pores within the porous elastomeric polymer, the blue region was identified as the elastomeric polymer, and the dashed-line-framed area was identified as the overall structure of the porous elastomeric polymer. The porosity was defined as the ratio of the total area of the white circular region to the area of the porous elastomeric polymer within the dashed box. The material parameters selected for the study of the structural properties of the elastomeric polymer were 3 MPa for Young’s modulus, 0.49 for Poisson’s ratio, and 2.75 for the relative dielectric constant. Four distinct models were established based on various structural factors, including porosity, pore radius, major-to-minor axis ratio of elliptical pores, and rotation angle of elliptical pores, as depicted in Figure 1b (Groups 1–4). Figure 1b (Group 1) depicts three distinct dielectric layer structures with porosities of 10%, 40%, and 70%. The porosity of the elastomeric polymer was adjusted by changing the size of the pores while maintaining a constant number of pores. In Figure 1b (Group 2), the radii of the pores in the elastomeric polymer were 30 μm, 80 μm, and 250 μm. These pores with varying radii were achieved by adjusting the number of pores while maintaining the same porosity level. Figure 1b (Group 3) illustrates the structure of elliptical pores with a major-to-minor axis ratio of 1.2:1, 1.5:1, and 2:1. This structure was achieved by adjusting the aspect ratio of the major and minor axes while keeping the pore area constant. The structural models depicted in Figure 1b (Group 4) are presented for the cases where the rotation angle of the elliptical pore’s major axis was 45°, 60°, and 90°. Boundary conditions were defined as follows: pressure and terminal voltage boundary conditions were imposed on the top electrode, while fixed and ground boundary conditions were applied to the bottom electrode. Moreover, the mesh was adequately partitioned to guarantee a minimum resolution of 4 at the narrowest point between the pores.

### 2.2. Preparation of Porous PDMS Dielectric Layer

**Figure 2:**
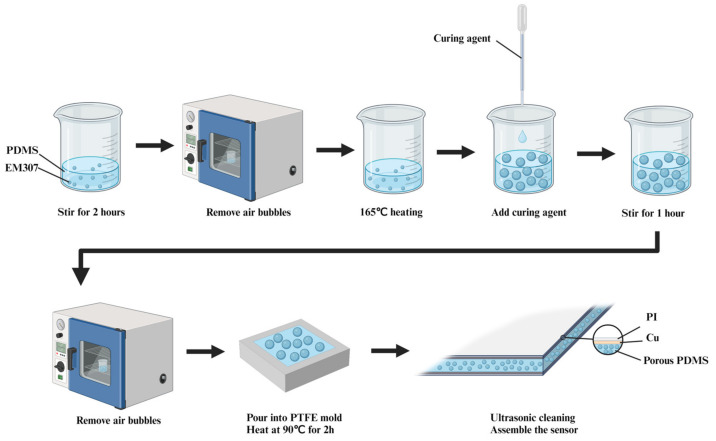
The schematic diagram of the preparation process for porous PDMS.

As shown in Figure 2, the procedure for preparing the PDMS porous dielectric layer involved the following steps: Initially, thermal expansion microspheres (EM307, Sekisui, Osaka, Japan) were added to PDMS precursors (Sylgard 184, Dow Corning, Midland, MI, USA) and stirred for two hours to ensure homogeneous mixing. The mass fractions of the thermal expansion microspheres were 1 wt%, 2 wt%, 2.8 wt%, and 3 wt%. The mixture was placed in a vacuum oven to eliminate bubbles and was subsequently heated at 165 °C for 12 min. During this process, thermal expansion microspheres expanded due to the effect of heat. Subsequently, the curing agent was added to the mixture and stirred for one hour. After being stirred, the mixture was placed in the vacuum oven to eliminate bubbles. The ratio of PDMS precursors to curing agent was 10:1. The mixture was subsequently transferred into a polytetrafluoroethylene (PTFE) mold for curing. After the curing process was complete, porous PDMS was subjected to ultrasonic cleaning with a solution of dimethylformamide (DMF). Finally, copper foil tape was applied to adhere to the surface of the porous PDMS as the electrode layer, and polyimide (PI) tape was used as the insulating layer, completing the assembly of the sensor.

## 3. Results and Discussion

### 3.1. The Impact of Porous Elastomeric Polymer Structure on Sensor’s Sensitivity

#### 3.1.1. Porosity

The porosity of the elastomeric polymer plays a crucial role in regulating the sensor’s sensitivity. During the compression process, the porosity gradually decreased, leading to a change in the dielectric constant. Consequently, we propose a more rigorous derivation of the sensitivity–porosity relationship.

**Figure 3:**
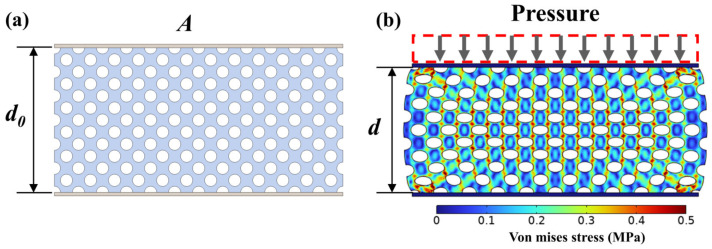
The compression process of porous flexible capacitive pressure sensors. (**a**) The sensor model prior to compression. (**b**) The stress nephogram of sensor after compression.

As illustrated in Figure 3a, if the surface area of the top electrode is denoted as A, and the height of the dielectric layer before compression is $d_{0}$, the initial capacitance can be determined as follows:

(1) $$C_{0}=\frac{\varepsilon_{0}\varepsilon_{e}A}{d_{0}}+C_{f}$$

where $\varepsilon_{0}$ represents the vacuum dielectric constant, $\varepsilon_{e}$ signifies the relative dielectric constant of the porous dielectric layer, and $C_{f}$ denotes the capacitance attributed to the induced edge effect. The relative dielectric constant of the porous dielectric layer can be expressed by the following formula:

(2) $$\varepsilon_{e}=\varepsilon_{\mathrm{air}}\varphi +\varepsilon_{\mathrm{polymer}}(1-\varphi )$$

where $\varepsilon_{\mathrm{air}}$ represents the relative dielectric constant of air, $\varepsilon_{\mathrm{polymer}}$ denotes the relative dielectric constant of elastomeric polymer, and φ stands for the porosity. Porous elastomeric polymer undergoes deformation when external pressure is applied. Figure 3b shows the stress nephogram after compression. During the compression process, it was evident that circular pores were flattened, approximating an elliptical shape. Moreover, in the central region of the porous elastomeric polymer, deformation was more pronounced, leading to a more evident concentration of stress. If we assume that the height of the compressed porous elastomeric polymer is d, the capacitance changes can be expressed as follows:

(3) $$\Delta C=C-C_{0}=\frac{\varepsilon_{0}\varepsilon_{e1}A}{d}-\frac{\varepsilon_{0}\varepsilon_{e}A}{d_{0}}$$

where $\varepsilon_{e1}$ represents the relative dielectric constant of the compressed porous dielectric layer and can be expressed by the following equation:

(4) $$\varepsilon_{e1}=\varepsilon_{\mathrm{air}}\varphi_{1}+\varepsilon_{\mathrm{polymer}}(1-\varphi_{1})$$

where $\varphi_{1}$ stands for porosity after compression. The volume change in the dielectric layer was approximately equivalent to the product of the displacement caused by the compression and the area of the top electrode. As depicted in Figure 3b, the volume within the dashed outline corresponds to the volume alteration of the porous dielectric layer. Therefore, the porosity after compression is given by Equation (5):

(5) $$\varphi_{1}=\frac{Ad_{0}\varphi -A(d_{0}-d)}{Ad}=\frac{d_{0}}{d}\varphi -\frac{d_{0}}{d}+1$$

By substituting Equations (4) and (5) into Equation (3), the capacitance changes can be expressed as follows:

(6) $$\Delta C=\frac{\varepsilon_{0}A[\varepsilon_{\mathrm{air}}(\frac{d_{0}}{d}\varphi -\frac{d_{0}}{d}+1)+\varepsilon_{\mathrm{polymer}}(-\frac{d_{0}}{d}\varphi +\frac{d_{0}}{d})]}{d}-\frac{\varepsilon_{0}\varepsilon_{e}A}{d_{0}}$$

The sensitivity of the sensor is correlated with the relative changes in capacitance and pressure variation ΔP, and can be expressed as follows:

(7) $$S=\frac{\Delta C}{\Delta PC_{0}}$$

Substituting Equation (6) and Equation (1) into Equation (7) gives the sensitivity formula, as follows:

(8) $$S=\frac{d_{0}\varepsilon_{0}A[\varepsilon_{\mathrm{air}}(\frac{d_{0}}{d}\varphi -\frac{d_{0}}{d}+1)+\varepsilon_{\mathrm{polymer}}(-\frac{d_{0}}{d}\varphi +\frac{d_{0}}{d})]}{\Delta Pd(\varepsilon_{0}\varepsilon_{e}A+d_{0}C_{f})}-\frac{\varepsilon_{0}\varepsilon_{e}A}{\Delta P(\varepsilon_{0}\varepsilon_{e}A+d_{0}C_{f})}$$

**Figure 4:**
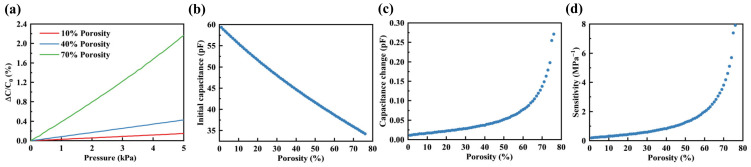
The impact of porosity on the sensitivity of the sensor. (**a**) The relative capacitance changes at porosities of 10%, 40%, and 70% within the range of 0 to 5 kPa. (**b**) The initial capacitance across porosities ranges from 1% to 76%. (**c**) The capacitance changes under a 1 kPa applied pressure across a range of porosities from 1% to 76%. (**d**) The sensor’s sensitivity varied within the range of porosities from 1% to 76%.

The FEM results depicted in Figure 4a demonstrate the relative capacitance changes of sensors with three distinct porosities (low: 10%, medium: 40%, high: 70%) within the 0 to 5 kPa range. The relative capacitance changes exhibited a linear growth trend with the increasing pressure, and the slope of this trend increased in correlation with the porosity of the porous elastomeric polymer. The initial capacitance, capacitance changes under 1 kPa pressure, and sensitivity were obtained by the FEM within the porosity range of 1% to 76% in the porous elastomeric polymer, as illustrated in Figure 4b–d. The observed trend indicates that as the porosity of the porous elastomeric polymer increased, the initial capacitance of the sensor linearly decreased. The decrease in the relative dielectric constant of the porous elastomeric polymer was directly proportional to the increasing porosity. This led to a decrease in the initial capacitance, as demonstrated in Equations (1) and (2). Moreover, as porosity rose, there was a corresponding increase in capacitance changes. The increase in porosity led to a decrease in the modulus of the porous elastomeric polymer. Under identical levels of pressure, the deformation escalated, resulting in an increase in the capacitance changes. Consequently, the sensitivity increased as the porosity of the porous elastomeric polymer increased. The influence of the porosity of porous elastomeric polymers on the sensitivity of sensors was significant. The sensitivity increased by a factor of 42 compared with the sensitivity at 1% porosity, when the porosity level reached 76%. Furthermore, as porosity increased, the sensitivity demonstrated an exponential growth trend. In the high porosity range, especially when it exceeded 60%, porosity significantly impacted sensitivity. For instance, when the porosity was at 76%, the sensitivity was approximately four times higher than that at 60% porosity. In contrast, the sensitivity at a porosity of 60% was only twice as high as that at a porosity of 44%. In summary, the adjustment of the porosity of the elastomeric polymer impacted both the initial capacitance and the capacitance changes of the sensor, consequently modifying its sensitivity.

#### 3.1.2. Pore Radius

**Figure 5:**
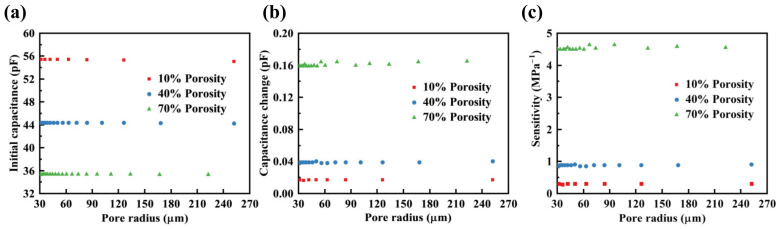
Effects of pore radius on the sensitivity of the sensor. (**a**) Initial capacitance for different pore radii in the range of 30 to 250 μm under three porosity levels (10%, 40%, 70%). (**b**) Capacitance changes under 1kPa pressure for different pore radii in the range of 30 to 250 μm across three porosity (10%, 40%, 70%). (**c**) Sensitivity for different pore radii in the range of 30 to 250 μm under three porosity levels (10%, 40%, 70%).

During the fabrication process of the dielectric layer, non-uniform pore sizes may be encountered. A series of porous elastomeric polymers with pore radii ranging from 30 to 250 μm and porosities of 10%, 40%, and 70% were selected. The initial capacitance, capacitance changes under a pressure of 1 kPa, and sensitivity were obtained by the FEM, as illustrated in Figure 5a–c. It was observed that with an increase in pore radius, the initial capacitance, capacitance changes, and sensitivity remained constant. This trend remained constant across porosities of 10%, 40%, and 70%. The results indicate that changing the size of the pores had little impact on the sensitivity of the sensor when the porosity remained constant.

#### 3.1.3. Major-to-Minor Axis Ratio

**Figure 6:**
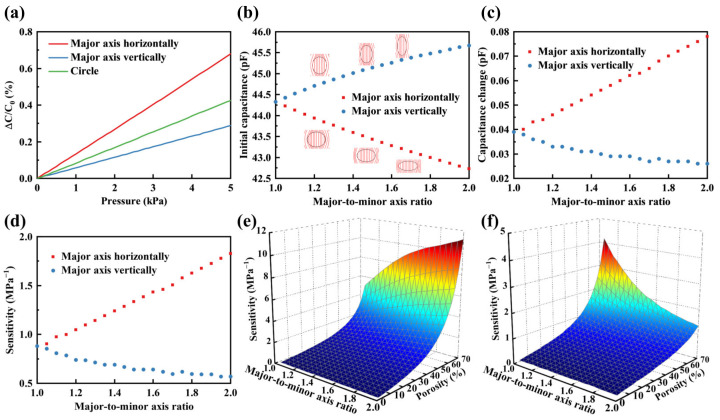
Effects of the major-to-minor axis ratio on the sensitivity of sensor. (**a**) Relative capacitance changes for elliptical pores with horizontal major axis, vertical major axis, and circular pores under a pressure range of 0 to 5 kPa. (**b**) Initial capacitance for elliptical pores with horizontal and vertical major axes at different major-to-minor axis ratios. (**c**) Capacitance changes under 1 kPa pressure for elliptical pores with horizontal and vertical major axes at different major-to-minor axis ratios. (**d**) Sensitivity for elliptical pores with horizontal and vertical major axes at different major-to-minor axis ratios. (**e**) Three-dimensional (3D) plot comparing the effects of the major-to-minor axis ratio and porosity on sensitivity when the major axis was horizontal. (**f**) 3D plot comparing the effects of the major-to-minor axis ratio and porosity on sensitivity when the major axis was vertical.

The shape of the pores in the porous elastomeric polymer plays a crucial role in regulating the sensor’s sensitivity. Initially, three distinct porous elastomeric polymer structures were compared: those with horizontal elliptical pores, those with vertical elliptical pores, and those with circular pores. The porosity of the elastomeric polymer was 40%, characterized by elliptical pores with a major-to-minor axis ratio of 1.5:1. Relative capacitance changes of the sensors within the pressure range of 0 to 5 kPa by the FEM are illustrated in Figure 6a. The results indicate that, similar to circular pores, the relative capacitance changes of elliptical pores in the range of 0 to 5 kPa also exhibited a linear trend. Among the porous structures considered, major-axis horizontal elliptical pores demonstrated greater sensitivity compared with major-axis vertical elliptical pores and circular pores. Circular pores exhibited higher sensitivity compared with vertical elliptical pores with major axes. Subsequently, the impact of the major-to-minor axis ratio of the elliptical pore on the sensor’s sensitivity was investigated, with a specific focus on two scenarios: when the major axis was oriented horizontally and when it was orientated vertically. The initial capacitance, capacitance changes under 1 kPa pressure, and sensitivity were obtained by the FEM, as illustrated in Figure 6b–d. In scenarios where the major axis was horizontal, as the ratio increased there was a decrease in the initial capacitance and an increase in the capacitance changes. Consequently, the sensor’s sensitivity to pressure increased. Conversely, when the major axis was vertical, an increase in the ratio led to a rise in the initial capacitance while decreasing the capacitance changes. This led to a decrease in the sensor’s sensitivity to pressure. Moreover, it should be noted that sensitivity showed a linear increase with the major-to-minor axis ratio when the major axis was oriented horizontally. The sensitivity decreased as the major-to-minor axis ratio increased when the major axis was oriented vertically, and the rate of this decrease gradually diminished.

The electric field lines exhibited deformation due to the presence of pores within the elastomeric polymer, as illustrated in Figure 6b. When the major axis was oriented horizontally, an increase in the ratio of the major axis to the minor axis intensified the distortion of electric field lines, resulting in a decrease in the initial capacitance. Conversely, when the major axis was vertical, an increase in the major-to-minor axis ratio reduced the curvature of electric field lines, which resulted in a higher initial capacitance. Moreover, when the major axis was oriented horizontally, an increase in the major-to-minor axis ratio led to a decrease in the modulus of the porous elastomeric polymer. Under the same applied pressure conditions, this caused a more pronounced displacement of the top electrode, leading to increased variations in capacitance. When the major axis was oriented vertically, an increase in the major-to-minor axis ratio leads to an increase in the modulus of the porous elastomeric polymer. This, in turn, led to a reduction in the displacement of the top electrode and a decrease in the capacitance changes under the same applied pressure. Subsequently, an investigation was conducted on the collective influence of the major-to-minor axis ratio and porosity on the sensor’s sensitivity, as illustrated in Figure 6e,f. In scenarios where the major axis was oriented horizontally, the sensor’s sensitivity increased with higher porosity and a greater major-to-minor axis ratio. The sensitivity demonstrated an approximately linear growth trend as the major-to-minor axis ratio increased, with the slope gradually steepening as porosity increased. When the major axis was oriented vertically, sensitivity increased with greater porosity and a decreased major-to-minor axis ratio. As the major-to-minor axis ratio increased, the sensitivity decreased. It is noteworthy that as porosity increased, the rate of decline also increased.

#### 3.1.4. Rotation Angle of the Major Axis

**Figure 7:**
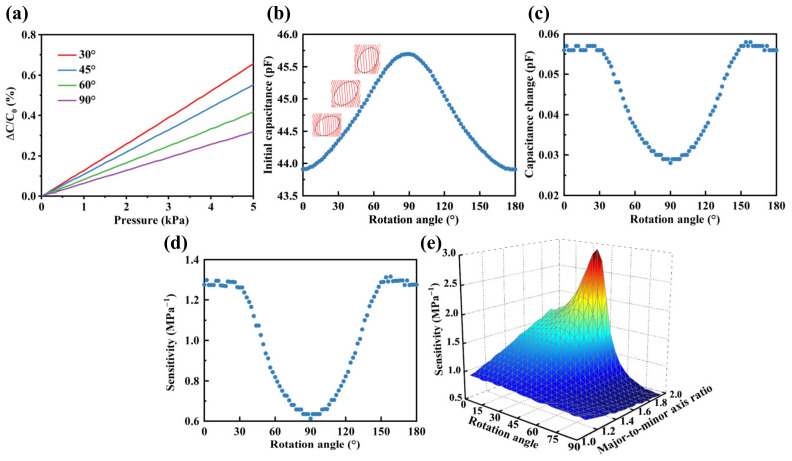
Effects of the rotation angle of the major axis on the sensitivity of the sensor. (**a**) Relative capacitance changes at various rotation angles (30°, 45°, 60°, 90°) within a range of 0 to 5 kPa. (**b**) Initial capacitance at different rotation angles within a range of 0° to 180°. (**c**) Capacitance changes at different rotation angles within a range of 0° to 180°under a pressure of 1 kPa. (**d**) Sensitivity at different major axis rotation angles within a range of 0° to 180°. (**e**) 3D plot comparing the effects of rotation angle and the major-to-minor axis ratio on sensitivity.

The orientation angle of the major axis of elliptical pores within porous elastomeric polymers significantly influenced the sensor’s sensitivity. The relative capacitance changes for major axis rotation angles of 30°, 45°, 60°, and 90° are illustrated in Figure 7a. In the pressure range of 0 to 5 kPa, it is apparent that the relative capacitance changes for various major axis rotation angles exhibit a linear trend. Moreover, with an increased rotation angle of the major axis, the slope of the relative capacitance changes decreases, and the sensitivity decreases. Subsequently, with a porosity of 40% in an elastomeric polymer, the initial capacitance, capacitance changes under a pressure of 1 kPa, and sensitivity were obtained using the FEM, as shown in Figure 6b–d. The initial capacitance increased in an S-shaped curve as the rotation angle ranged from 0° to 90° and exhibited a symmetrical trend between 0° to 90° and 90° to 180°. As depicted in Figure 7b, an increase in the rotation angle reduced the curvature of electric field lines and increased the initial capacitance. This phenomenon bears resemblance to the influence noted in the preceding section regarding variations in major-to-minor axis ratios. Moreover, within the range of 0° to 90°, an increase in the rotational angle led to an increase in the modulus of the porous elastomeric polymer. Consequently, the deformation of the porous elastomeric polymer decreased under the same applied pressure, resulting in a reduction in capacitance changes, as illustrated in Figure 7c. This contributed to the decrease in sensitivity, as depicted in Figure 7d. Furthermore, within the range of 0° to 30°, sensitivity remained relatively stable. However, beyond 30°, there was a noticeable decrease in sensitivity. Finally, an investigation was conducted into the collective impacts of rotation angle and major-to-minor axis ratio on sensitivity, as depicted in Figure 7e. Within the range of rotation angles from 0° to 60°, an increase in the major-to-minor axis ratio resulted in an improvement in sensitivity. Nevertheless, the increase in the major-to-minor axis ratio led to a reduction in sensitivity beyond a rotation angle of 60°. Additionally, when the major-to-minor axis ratio exceeded 1.7, sensitivity initially rose to a peak, followed by a steep decline, and finally reached a stable state with an increase in the rotation angle. Typically, this peak was observed between 30° and 40°.

### 3.2. The Influence of Material Property of Elastomeric Polymer on the Sensor’s Sensitivity

#### 3.2.1. Relative Dielectric Constant of Elastomeric Polymer 

**Figure 8:**
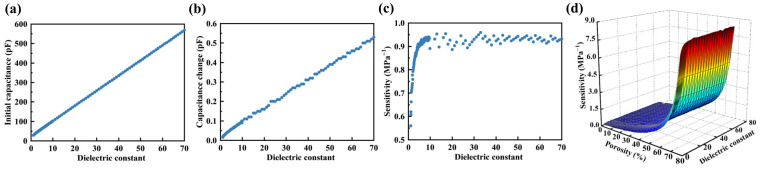
Influence of the relative dielectric constant of elastomeric polymer on the sensor’s sensitivity. (**a**) Impact of relative dielectric constant on initial capacitance within the range of 1 to 70. (**b**) Capacitance changes within the range of relative dielectric constant from 1 to 70 under a pressure of 1 kPa. (**c**) Sensitivity within the range of relative dielectric constant from 1 to 70. (**d**) 3D plot comparing the effects of porosity and dielectric constant on the sensor’s sensitivity.

The relative dielectric constant plays a crucial role in influencing the sensitivity of the sensor. To investigate the impact of the relative dielectric constant on the sensitivity of a porous flexible capacitive pressure sensor, an elastomeric polymer with a porosity of 40% and Young’s modulus of 3 MPa was chosen as the dielectric layer. Within the range of relative dielectric constants from 1 to 70, the initial capacitance, capacitance changes under 1 kPa pressure, and sensor’s sensitivity were obtained through the FEM, as illustrated in Figure 8a–c. The results indicate that the initial capacitance and capacitance changes increased linearly with the increase in the relative dielectric constant. However, sensitivity exhibited different trends with an increase in the relative dielectric constant. Sensitivity underwent a rapid increase when the relative dielectric constant was less than 5. When the relative dielectric constant exceeded 5, the rate of sensitivity increase gradually slowed down. Once the relative dielectric constant exceeded 10, sensitivity remained almost constant. Furthermore, the combined impact of porosity and the relative dielectric constant of elastomeric polymers on the sensor’s sensitivity was investigated, as illustrated in Figure 8d. The results indicate that higher porosity and relative dielectric constant contributed to increased sensitivity.

#### 3.2.2. Modulus of Elastomeric Polymer

**Figure 9:**
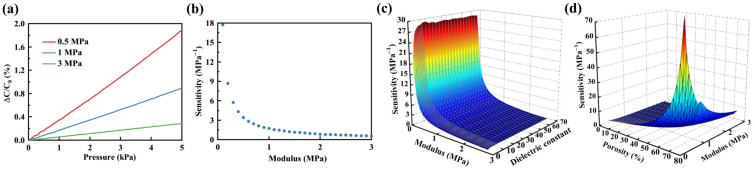
Effect of modulus of elastomeric polymer on the sensitivity of the sensor. (**a**) Within the pressure range of 0 to 5 kPa, the relative capacitance changes of a sensor with porous elastomeric polymer having Young’s modulus of 0.5 MPa, 1 MPa, and 3 MPa. (**b**) Influence of Young’s modulus of elastomeric polymer within the 0.1 to 3 MPa range on sensitivity. (**c**) 3D plot comparing the effects of Young’s modulus and dielectric constant of elastomeric polymer on sensitivity. (**d**) 3D plot comparing the effects of porosity and Young’s modulus of elastomeric polymer on sensitivity.

The modulus of the elastomeric polymer plays a crucial role in influencing the sensor’s sensitivity. Elastomeric polymers exhibit varying degrees of deformation under identical pressure levels due to differences in their moduli. Therefore, the sensor’s sensitivity can be adjusted by modifying the modulus. The relative capacitance changes of the sensor obtained through the FEM within the pressure range of 0 to 5 kPa are illustrated in Figure 9a. The elastomeric polymer used in the sensor had a porosity of 40% and moduli of 0.5 MPa, 1 MPa, and 3 MPa. The result revealed a linear increase in relative capacitance changes with rising pressure. Additionally, the sensitivity of the sensor diminished as the modulus increased. The relationship between the modulus and the sensor’s sensitivity obtained through the FEM within the range of 0.1 to 3 MPa is illustrated in Figure 9b. The results indicate that as Young’s modulus increased, the sensitivity of the sensor decreased, and the rate of decrease gradually slowed down. Additionally, the impact of modulus on sensitivity was particularly pronounced in the low modulus range (<1 MPa).

Subsequently, using the FEM, a comparative analysis was conducted to investigate the impact of the modulus and the relative dielectric constant on the sensor’s sensitivity, as depicted in Figure 9c. Sensitivity increased with a low modulus and a high relative dielectric constant. Finally, the combined effect of the porosity and modulus of the elastomeric polymer obtained through the FEM on the sensor’s sensitivity is shown in Figure 9d. It can be observed that both factors significantly influenced sensitivity. High sensitivity was achieved under conditions of high porosity and low elastomeric polymer modulus.

### 3.3. Experimental Results Analysis of Sensors with PDMS as the Dielectric Layer

#### 3.3.1. Impact of Different Modulus of PDMS on Sensor’s Sensitivity

**Figure 10:**
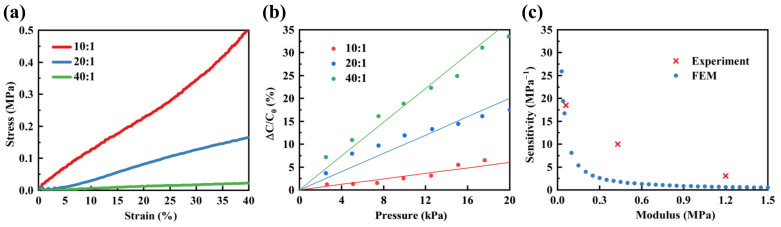
The impact of different curing agent ratios on sensor sensitivity. (**a**) The stress–strain curves of PDMS under three different curing agent ratios of 10:1, 20:1, and 40:1. (**b**) The relative capacitance changes of the sensor under three different curing agent ratios of 10:1, 20:1, and 40:1. (**c**) The sensitivity of the sensor under different modulus conditions.

Modifying the ratio of PDMS to curing agent allowed for the adjustment of PDMS modulus, consequently impacting the sensor’s sensitivity. In this section, PDMS-to-curing agent ratios of 10:1, 20:1, and 40:1 were chosen to fabricate PDMS samples with different moduli. Stress–strain tests were conducted, as illustrated in Figure 10a, and Young’s modulus of PDMS under different curing agent ratios is presented in Table 1.

Subsequently, PDMS with different ratios of curing agent was employed as the dielectric layer for sensors, and the relative capacitance changes were measured, as illustrated in Figure 10b. The results indicate that a higher sensitivity was noted with a lower curing agent content. Finally, an investigation was conducted on the relationship between the modulus of PDMS and the sensor’s sensitivity. The comparative results between the experiments and the FEM are shown in Figure 10c. It is apparent that sensor’s sensitivity diminished as the modulus increased, with the rate of decline gradually decelerating.

#### 3.3.2. The Impact of the Porosity of Porous PDMS on the Sensitivity of Sensors 

**Figure 11:**
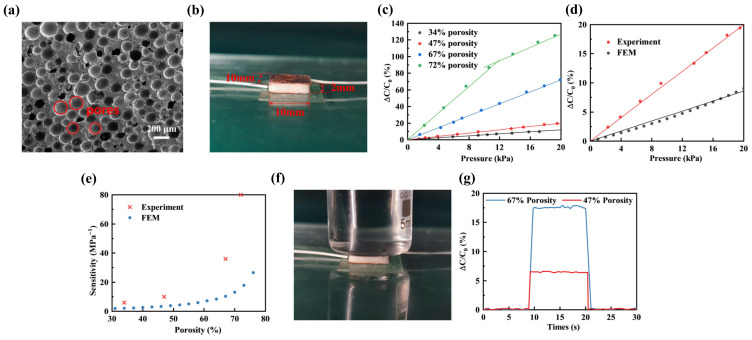
Impact of the porosity of porous PDMS on the sensitivity of sensors (**a**) SEM image of the porous PDMS with 2 wt% thermal expansion microspheres. (**b**) Photograph of the porous flexible capacitive pressure sensor with porous PDMS as the dielectric layer. (**c**) Relative capacitance changes of the sensor at porosities of 34%, 47%, 67%, and 72% within the pressure range of 0 to 20 kPa. (**d**) Comparison of experimental and FEM results for relative capacitance changes of a sensor with 47% porosity porous PDMS as the dielectric layer in the range of 0–20 kPa. (**e**) Comparison of experimental and FEM sensitivity results at different porosities. (**f**) Photograph of a beaker filled with water placed on the sensor. (**g**) Relative capacitance changes when a beaker filled with water was placed on sensors with porosities of 67% and 47%.

We adjusted the porosity of porous PDMS sensors by incorporating varying mass fractions of thermal expansion microspheres. The porosity was determined by the formula:

(9) $$f=1-\frac{m}{M}$$

where f represents the porosity, m corresponds to the mass of the prepared porous PDMS, and M denotes the mass of bulk PDMS with the same volume. Figure 11a shows the scanning electron microscope (SEM) image of a porous PDMS with 2 wt% thermal expansion microspheres. The red circular markers indicate the prepared pores, which exhibited an approximately uniform distribution in the PDMS. The pore diameter was approximately 180 μm. The photograph of the sensor is shown in Figure 11b, with dimensions of 10 mm in both length and width, and a height of 2 mm. In the pressure range of 0 to 20 kPa, we tested the relative capacitance changes of the sensors using four different porous PDMS with porosities of 34%, 47%, 67%, and 72% as the dielectric layers, as illustrated in Figure 11c. In the range of 0 to 20 kPa, the relative capacitance changes of the sensor exhibited an approximately linear increase with pressure for porosity levels of 34%, 47%, and 67%. When the porosity reached 72%, the sensor exhibited higher sensitivity within the range of 0 to 10 kPa. However, beyond 10 kPa, the slope of the relative capacitance changes decreased. This was attributed to the squeezing of pore walls, resulting in an increased modulus and consequently a reduction in slope. Subsequently, the FEM was performed utilizing the simulation model described in Section 2.1 (Group 1). In this model, the white circular regions represent the porous PDMS pore structures. The model utilized an array distribution to approximate the uniform distribution of samples. Additionally, the Young’s modulus of PDMS was experimentally measured at a PDMS-to-curing agent ratio of 10:1, as outlined in Section 3.3.1, serving as material parameters for the FEM. Porous PDMS with a porosity of 47% was chosen as the dielectric layer. Within the range of 0–20 kPa, a comparison was conducted between the experimental test results and the FEM results of the relative capacitance changes, as illustrated in Figure 11d. The result indicates that the sensor demonstrated satisfactory linearity within the 0–20 kPa range. However, the experimental results showed a relatively steeper slope in comparison to the FEM results. Additionally, the sensitivity of the prepared samples within the 0–10 kPa range was calculated and compared with FEM results, as illustrated in Figure 11e. It was observed that with an increase in porosity levels, there was a corresponding increase in the sensor’s sensitivity, particularly within the high porosity range, where the impact of porosity on sensitivity was more pronounced. The experimental results exhibited a similar trend to the FEM results. However, the sensitivity measured in the experimental results was higher than that obtained from the FEM. The disparity between experimental outcomes and the FEM results was primarily due to the difference between the 2D model and the experimental sample, such as the array distribution employed in the 2D model being different to the random distribution present in the experimental sample, leading to disparities between the FEM and the actual measurement outcomes. Then, we choose PDMS with a porosity of 67% and 47% as the dielectric layer for the sensor and placed a beaker filled with water on top of the sensor, as shown in Figure 10e. It can be seen that the relative capacitance changes of the two sensors were different. The relative capacitance changes of the porosity of 67% was higher than that of 47%, as shown in Figure 11f.

Finally, a comparison was conducted between the porous PDMS sensor fabricated in this study and previous research on flexible capacitive pressure sensors, as presented in Table 2. It can be observed that the sensor that we prepared demonstrated higher sensitivity.

## 4. Conclusions

In summary, this study investigated the application of porous elastomeric polymer dielectric materials in flexible pressure sensors and proposes a method to modify the sensor’s sensitivity by adjusting the parameters of the elastomeric polymer. The FEM was employed to investigate the impact of pore structural characteristics and material properties of the porous elastomeric polymer on the sensor’s sensitivity. Among the structural characteristics of pores, porosity played a predominant role in influencing sensitivity, particularly within the high range of porosity (exceeding 60%). For example, when the porosity reached 76%, the sensitivity increased approximately fourfold in comparison to that at 60% porosity. The sensitivity of the sensor was further influenced by the ratio of the major-to-minor axis of the elliptical pore. When the major axis was oriented horizontally, sensitivity exhibited a linear increase with the ratio. When maintaining a constant porosity level, a ratio of 2:1 resulted in a sensitivity that was twice as high as that of a circular pore. Nevertheless, pore size and the rotation angle of elliptical pores had relatively minor effects. The Young’s modulus of the elastomeric polymer significantly influenced sensitivity, particularly when it was below 1 MPa. Additionally, a higher dielectric constant (less than 10) was beneficial for improving the sensitivity of the sensor. Porous PDMS with varying porosities was fabricated as the dielectric layer through the thermal expansion microsphere method. A comparison of the sensors’ sensitivity across various porosities was conducted. The sensitivity at 47% porosity was approximately 1.6 times higher than that at 34%. The sensitivity at 72% porosity exhibited approximately twice the magnitude compared with that at 67% porosity.

## Figures and Tables

**Table 1:** The Young’s modulus of PDMS under varying proportions of curing agents.

10:1	20:1	40:1
1.2 MPa	0.43 MPa	0.06 MPa

**Table 2:** Comparison of sensitivity and detection range of capacitive pressure sensors.

Reference	Dielectric Material	Dielectric Structure	Maximum Sensitivity (MPa^−1^)	Working Pressure (kPa)
[34]	PDMS	Porous	23	0–200
[25]	PDMS	Porous	10.7	0–12
[35]	PDMS	Solid	1.6	0–945
[36]	PDMS	Micro-array	32	0–50
[37]	PDMS/GO	Solid	2	0–400
[38]	Ecoflex/PDMS	Microbeads	48	0–10
[39]	PDMS/Ag@CNTs/AgNWs	Solid	49	0–45
[40]	PDMS	Pyramid	34	0–100
This work	PDMS	Porous	80	0–10

## Data Availability

Data can be obtained from the authors on request.
